# Monica Healthcare: From the research laboratory to commercial reality—A real‐life case study

**DOI:** 10.1049/htl2.12004

**Published:** 2021-02-23

**Authors:** Barrie R Hayes‐Gill

**Affiliations:** ^1^ Faculty of Engineering University of Nottingham Nottingham UK

## Abstract

The desire of many engineers is to see their work end up as a final product offering a real benefit to society—for a lecturer/professor at a university, this is a dream often out of reach of the majority. However, the university academic is a changed species from the early days of the binary line between Universities and Polytechnics and when a lecturer meant just that—teaching to future engineers. This article describes the process and experience gained by a university engineer to spin out their research from the university sector and achieve the goal of a product reaching a global audience.

## 1 INTRODUCTION

It was 1986 when I started my academic role at the University of Nottingham (UoN). Within one month, my wife—who had just given birth to our second child—was diagnosed with an aggressive cancer called adenocarcinoma. Tragically, she died after 12 months. It was horrendous but the university was fantastic and told me to look after my family and put my research on hold for a bit. When I returned to work, I was understandably more interested in healthcare research and how electronics could help.

A short time later, a highly respected colleague also died of cancer—Professor Derek Kirk. He had been undertaking research into foetal monitoring. I went to see him in hospital, where he suggested carrying on his work with other colleagues in my department. These two significant deaths in my life led me to foetal monitoring as an area of my research.

Having now spent over 30 years as an electronic engineer at the UoN, it is fair to say that the landscape for the academic researcher has changed out of all recognition. The word “academic” is now a distant definition of a professional university engineer, who is now driven by research league tables to create impact in society and even employment for the benefit of all. It is, therefore, no surprise that to do this means looking beyond solving the micro‐level challenges of the laboratory and making connections, with external professionals who can help refine your discoveries, and with industry leaders in order to commercialise and scale up the product or process.

My journey has resulted in over 25 patents filed with colleagues in the Optics and Photonics Research Group at UoN and jointly securing more than £20m funding and investment from commercial and public sector partners. These are outputs that you can never achieve on your own and the importance of the team is vital.

A significant highlight has been the research, development and technology transfer of a wearable wireless foetal and maternal care medical device. This novel technology formed the basis of a spin‐out company, Monica Healthcare, to commercialise this university research. The collaboration with Philips Healthcare and the subsequent sale of Monica to GE Healthcare generated significant revenue for the university and speeded up the roll out of the technology now available to millions of women and unborn babies around the world.

This article sheds some light on the process by which an idea in a university laboratory found its way into the arms of both GEHC and Philips—two of the largest healthcare conglomerates in the world.

## 2 THE EXISTING TECHNOLOGY AND LIMITATIONS

So, it was in 1988 that myself and John Crowe (another academic engineer in our faculty) continued Prof Kirk's pioneering work on foetal monitoring. We set about pondering the challenge of improving the foetal monitors in hospital. In the 1960s and 1970s, many technologies for monitoring the foetus and the mother had been commercialised, including using a microphone (phonocardiography) and abdominal foetal electrocardiogram (fECG) by placing electrodes over the abdomen to pick up the foetal heart.

By the 1980s, the technology had focussed on just one solution using two transducers placed on a pregnant mother's abdomen—one for detecting a foetal heart beat (Doppler ultrasound) and the other for detecting mother's contractions (an abdominally placed strain gauge guard ring transducer called a tocodynamometer ‐ “TOCO”). By the 1990s, these two signals, one from the baby and one from the mother, had become crucial for detecting the well‐being of a foetus during labour. For example, when a uterine contraction occurs, the foetal heart rate (FHR) can drop as the blood supply to the foetus is diminished by the contracting uterus. The time it takes for the FHR to recover is an indication of foetal health and its ability to survive the rest of labour.

Both transducers (Doppler FHR and TOCO) are attached via an elasticated belt around the mother's abdomen and are called a cardiotocograph (CTG). This arrangement, although state of the art in 1990, presented several problems (and still does) such as:

- The belts used to secure the transducers are uncomfortable and need constant re‐adjustment by the attending team as the foetus moves lower in the uterus as labour progresses or these belts simply slip on the abdomen. If the transducers are not re‐positioned, errors in the monitored signal can creep in, often without the care‐team's knowledge.
- With many CTGs, the mother is effectively tied to the bed from the restricting belts, meaning that the obvious encouraging effect of gravity is not present during labour as efficacy studies has suggested [1].
- Ultrasound has problems penetrating fatty tissue, and hence, high‐body‐mass‐index (BMI) mothers have poor FHR detection resulting in the potential to miss or mask key FHR events [2].
- When the foetal and maternal heart beats, the Doppler ultrasound detects multiple mechanical movements of the foetal heart valves and heart walls as well as the blood flow both in the foetus and in the mother. This means that the Doppler signal can have many components depending on the position of the transducer and the presentation of the foetus. The problems here are that this can confuse the autocorrelation algorithm that calculates the FHR. Two beats can be detected, and hence, the highly problematic “doubling” in FHR can occur, i.e. a foetus whose FHR is 120 bpm (normal) can be shown as 240 bpm or worse still an FHR of 60 bpm (abnormal) can be portrayed as 120 bpm (normal). Or, in the case of maternal blood flow being erroneously detected, the heart rate can show 80 bpm, which is normal for the woman but very abnormal for the foetus or in the case of an exhausted mother can be above the FHR at say 160 bpm [3]. If the attending team are unaware of these potential problems, they can take no action when action is needed or take unnecessary action when the foetus is in fact well. In both cases (although rare ∼1/1000), there is potential for serious consequences to the mother or foetus.
- The contraction TOCO strain gauge is notoriously unreliable requiring careful positioning with the correct maternal belt tightening allowing reliable contraction detection.
- Finally (albeit recently), 50% or £2.4bn of NHS Resolution annual claims are from childbirth where the CTG is often cited as problematic, indicating that improvements were and are necessary [4].

Despite all these known problems, Doppler Ultrasound and tocodynamometry had become the standard of care for labouring women in the developed world. However, with these disadvantages, a possible opportunity existed for an improved product and with 9M births/year (EU and USA) of which 80% were monitored during antenatal and birth the market was there. Significantly, the only alternative to detect the FHR was to use the invasive method of a foetal scalp electrode that penetrated (by ∼1 mm) the foetal scalp. This was and remains the gold standard, but it can only be applied in the later stages of labour, and as one would expect, most women and clinicians do not like using it. There is also an invasive alternative to monitoring contractions using a pressure sensing tipped catheter inserted transvaginally into the uterus. Again, it can only be used in the later stages of the labour and is even less liked by clinicians and labouring women. The market was, therefore, ready for a safe and acceptable alternative to Doppler ultrasound and tocodynamometry.

## 3 INITIAL RESEARCH

We knew that when abdominal fECG was tried in the 1960s and 1970s, it worked well in some women, but not enough to make it a practical monitor, but it was a promising research area. So, we set off with the task of answering the question: Could electrophysiological monitoring on the abdomen of a pregnant mother detect both foetal heartbeats and contractions better than Doppler ultrasound and tocodynanometer?

**FIGURE 1:**
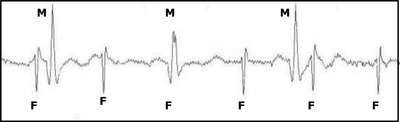
Time trace of an fECG (F) and maternal ECG (M) detected from the abdomen of a pregnant mother to be. Note the coincidence of F and M at one moment and also the noise present between each ECG complex. This is a particularly strong fECG signal

It was a wonderful challenge for an engineer to play with. A foetus's heartbeat electrocardiogram (ECG) on a good day on the mother's abdomen is around 10 μV (typically 3 μV), whereas the mother's heartbeat is between 1000 and 5000 μV on the sternum, whilst on the abdomen, it can be 200 μV compared to 3 μV for the foetal ECG (fECG) —a huge dynamic range if you want to successfully monitor both. Add electrical muscle and background electrical noise, and the isolation and detection of the tiny fECG signal is highly problematic. Figure 1 shows very clear signals detected on the abdomen illustrating fECG, maternal ECG and the noise.

We started our research work in 1990 with a series of PhD funding from the Engineering and Physical Sciences Research Council (EPSRC), the Collaborative Awards in Science and Engineering (CASE) scheme, the university, and the Malaysian government (Malaysian Royal Family and its PM were ex graduates of Nottingham University) in an attempt to build a foetal monitor that could detect these tiny signals. The Collaborative Awards in Science and Engineering scheme is excellent and involves an industrial partner adding a financial supplement to an EPSRC PhD award to work in a future theme for the company. We received financial support from our university research fund (£7800—predecessor of the current EPSRC Impact Accelerator Award) and the Royal Society (£5000) in 1992 and 1993, respectively. These were only small grants but vital as they allowed us to build the electronics, and we were very proud of our first FHR instrument in 1993. We presented this to the Hospital Clinical Engineering Department only to find out that our device failed BS5724 (a predecessor of BS60601), the electrical safety standard for medical devices. Although this was embarrassing, it was a fantastic lesson to our young medical devices group forcing us to become fully knowledgeable of the medical device safety standards. A skill‐base that has allowed us to design medical devices fit for purpose and taught us that understanding the medical devices regulation places us in a strong commercial position. For those of you not aware of the regulations surrounding medical devices, then, at first, the subject area is daunting especially as the EU Directive on medical devices (MDD93/42) had not really come into play at the time (1993), but it was evolving. Medical devices are graded based upon their level of risk as Class I (lowest risk), IIa, IIb and III (Invasive). Following the MEDDEV Guidance 2.4/1 rules for Classification of Active Devices, our device is classified as Class IIb. Anyone looking to commercialise their medical device must understand the associated medical devices law before placing a product on the market. Interestingly, the UK medical devices community having originally aligned to the EU MDD93/42 and its 2007 amendment were starting to understand the new EU regulations of MDR745. However, as we know, Brexit is having a big influence on regulatory matters, and at the time of writing, the new law for the UK for medical devices will be under the banner of “UKCA”.

We had built up a tremendous relationship with the Business Director from Oxford Medical, Dr. Terrence Martin, who was extremely knowledgeable on the existing Doppler technology and fully aware of its limitations—but at the time it was a case of Hobson's choice. Our technology offered an alternative although in 1996 our FHR detection rate was only 40%. To increase this, we applied (and received) an Action Medical Research (AMR) grant (£56,217) to build a portable FHR device.

We had a champion in Professor David James, an Obstetrician and Dean of the Medical School at Queen's Medical Centre (Nottingham), where trials and modifications increased the monitor's sensitivity and reliability to ∼60%. The application of the electrodes to a pregnant mother's abdomen was implemented by Karnie Bhogal a highly professional and knowledgeable NHS midwife who stayed with us throughout the later years of the project.

However, the required success rate (∼85%) needed for a commercial product was still a distant objective. One of our final foetal monitoring PhDs was a top French engineer (Jean Francois Pieri) visiting us on an Erasmus undergraduate program. We offered him a PhD place in 1997, and a delightful working relationship was developed during which we discovered a wonderful book by Henry Ott on noise reduction techniques and also the importance of an early morning coffee and croissants—the French way!

## 4 FURTHER RESEARCH

By 2000, most of our efforts had been placed on the signal processing, but if we could improve the quality of the raw abdominal signals, then extraction of the FHR would become easier. We picked up a useful pragmatic philosophy from National Semiconductor's analogue designers—“the problem is like an onion, remove one layer only to find another”. We recognised that perseverance and thoroughness was the key to achieving a successful instrument.

From 2000 to 2004, we made considerable research steps. I recall in 2001 the numerous researchers working on the signal processing of abdominal fECG data with moderate success. I constructed a pros and cons graphic of each research centre. Key parameters surfaced: fidelity of the detection instrument, no wireless link, a requirement for a leadless system, and finally no real‐time FHR extraction. These parameters, therefore, defined the high‐level specification of our design requirements.

By late 2000, we had filed our first patent (EP1220640B1, US7532923B1), completed one research programme grant from AMR and now employed Jean Francois (after his PhD) on a second AMR grant. The procedure at the time for filing patents within a university was considerably easier than it is nowadays. For example, we were required then to write a very short (one paragraph) description of our invention, estimate a market size and contact our equivalent of the technology transfer office (TTO) in those days called “Office of Research Business Services”. At this time, not many academics would file patents as there was no real requirement in the university league table metrics, whereas, now, the TTO is much more embedded within our Faculty of Engineering. Although there was budget available, it was not very competitive. Furthermore, since we had many projects funded under a CASE award, then often the company would pay for the initial UK filing fee.

Nowadays, the university internal processes require staff to submit an “IP Disclosure Form” containing: the problem, how the invention addresses this problem, its key advantages, the closest competitors, the prior art, a market analysis, which companies may be interested in this patent, etc. If initially approved by the University IP Commercialisation Office, then the inventors are required to present their invention to the Faculty IP committee, where you have the chance to “sell” your invention—somewhat like a “Dragons Den” but without the business route. Budgets are strictly controlled and typically the centre funds 66% and the faculty 33% but with the university metrics requirements for generating income from IP then spin outs and filing patents is often a necessity. Industrial partners are, therefore, often sought early so as to release IP positions when sensible. The ownership of the patent is the university; however, an agreed royalty sharing scheme is in place for the named inventors (and others) in the event of the patent being commercialised or equity can be given if a spin‐out is created as in our case. Once approved, the inventor will interact with a patent attorney using a hypothetical journal paper description of the technology, and from this, construct a draft specification sometimes with claims. The first stage is often to file in the UK, which sets an international “priority date”. An international Patent Co‐operation Treaty (PCT) application will be filed at 12 months with claims, and your patent is published at 18 months. It is at this point that you can submit your journal paper for publication as well—holding a journal paper back for these 12–18 months often frustrates academics, and it is possible to publish just after the priority date but you should speak to your patent attorney first or else you may lose your patent ! The PCT process secures the option of filing applications in a wide range of countries at a later date without the need to identify the countries of interest at the point of filing. It simplifies international patent filing and prosecution and defers costs. Typically, 18 months after the PCT has been filed, the application proceeds to the national phase. The examination process resulting in grant or rejection of the application occurs at the national phase and is carried out independently by each national patent office the application has been filed with. We mainly choose the EU and USA territories, but on occasions, we did go further afield (i.e. India, China, Japan etc.) if we felt the invention was of high value. The sequential acronym I like to recall in this patenting process is “FPG”—meaning Filed (priority date), Publish (12–18 months later), Granted (can be anytime from 4 years to 10 years!)

**FIGURE 2:**
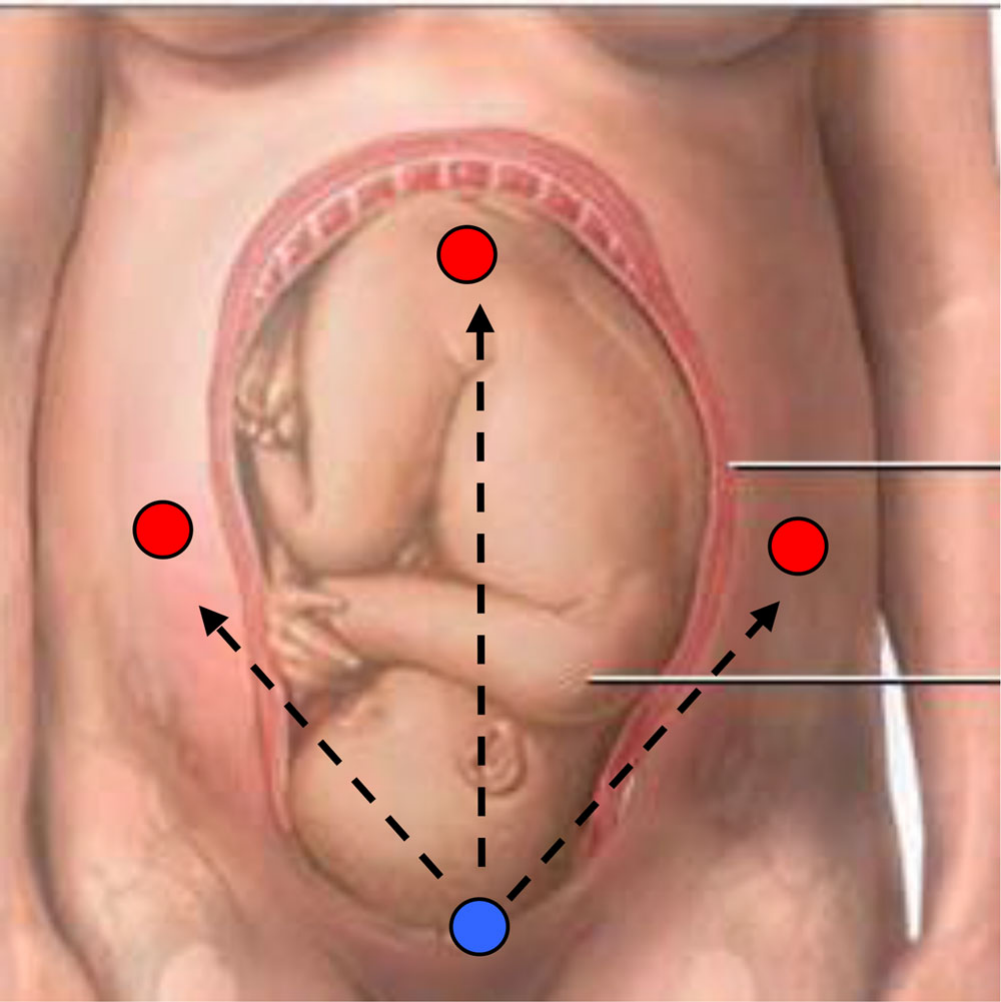
Three‐channel (red) fECG system proposed in 2001 with a common electrode (blue). As the foetus moves, the fECG can be seen on at least one channel [5]

**FIGURE 3:**
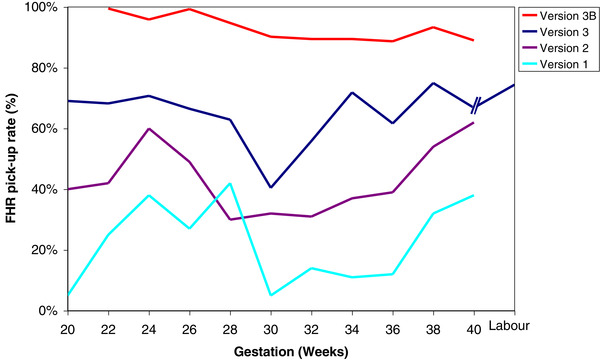
Increasing FHR detection rate as a function of gestation but with four improving versions of the electronic instrumentation showing the effect of reducing inherent electrical noise

A breakthrough came in the adoption of an abdominal three‐channel sensor (see Figure 2), along with a refined design of the electronics. We published these findings in 2001 [5]—little did we know that this paper would be cited on nearly 200 occasions by 2020. Here, we also demonstrated (see Figure 3) the increase in FHR detection with each version of the electronic instrumentation having reduced noise.

The three‐channel system presented us with another advantage. If the foetus moves out of the range of one channel, it falls into the range of another channel; this indicated movement—invaluable for monitoring foetal well‐being—and allowed us to construct a second patent in 2002 by which time we were achieving an FHR success rate of 70%. By now, we had over 500 recordings. We could see on each channel that the magnitude of the fECG signal varied as the foetus moved and often disappeared beneath the noise. However, when this happens, it would often appear on another channel; hence, combining this gave us the higher FHR detection rate. We again realised that the combination of several factors means that this is not a simple “one problem solved will fix all scenario”—our onion analogy.

Interestingly, we were approached in 2002 by a Doppler ultrasound CTG foetal monitoring company to validate their Doppler ultrasound FHR device in the antenatal period (i.e. when the mother's waters were still intact) against our abdominal three‐channel low‐noise fECG system. We collected raw fECG signals in parallel with Doppler ultrasound on 40 mothers at various gestations. We were, therefore, able to provide validation data for their CTG FHR algorithm, demonstrating that it matched our beat‐to‐beat three‐channel fECG data.

The significance of a Doppler CTG ultrasound company approaching us cannot be underestimated. It was an acceptance that our electrical detection method may well have real clinical benefit, and hence, an opportunity was appearing in front of our eyes. Our unique selling points (USPs) over belt‐based Doppler/TOCO were as follows:

- Simple electrodes adhering to mother's abdomen, i.e. no tight belts.
- Electrical signals are not affected by high BMI unlike Doppler ultrasound.
- The mother is no longer tied to the bed providing more maternal satisfaction and encouraging gravity to help with the birth process.
- Being a 100% passive electrical measurement opens itself to the benefits of wireless wearable technologies.
- The clear signature differentiation between a maternal ECG and an fECG dramatically reduces the possibility of MHR/FHR confusion.
- The uterus when contracting generates an electrical signal called the electrohysterogram (EHG). We had confirmed what other researchers had found that it was possible to detect contractions electrically—potentially sidestepping the unreliable and problematic tocodynamometer.
- With Moore's law on our side, we were able to neatly bundle the solution into a single‐wearable wireless flexible substrate (patch) potentially being simple to apply.

In 2003, we further optimised the FHR algorithm by introducing fECG signature recognition both in terms of duration and amplitude along with noise measurement allowing us to accurately separate out the signals from the noise. By 2004, our second patent [6] was finally filed. We spent the next 12 months trying to license the technology. Our process was to write a one‐page technology summary document and circulate via our University TTO to the Tier 1 foetal monitoring companies at the time. We had meetings with two of these companies and showed them our results, our device, and evidence of real recordings on over 500 pregnant mothers. However, we had no takers partly because: our product still had to be packaged correctly; we still only had a 70% FHR detection success rate; and we were considered a “me too” product. We quickly changed tack and secured small‐scale funding (HEFCE HIRF—a regional innovation fellowship scheme) of £15,900 to build a commercial team and develop a business plan. This highly flexible funding allowed us to recruit Dr. Carl Barratt (another of our ex PhD researchers), whose employer in Cambridge had folded—he was a highly focussed individual and little did we know that he would turn out to be a perfect CEO. We set about scoping the plan for a possible spin‐out. A JRC business plan success of £25,000 and a Medici Fellowship for Carl lay the foundations for this journey. This £25,000 business plan prize was highly welcome. Myself and Carl built up the business plan with the help of BioCity's experienced staff. The Lachesis Fund (University Challenge Seed Fund managed at the time by Quester) provided £15,000 for a due diligence confirmation that our two patents had value—we received not only a confirmation, but also the patent divisional law produced two more patents providing us with four patents.

## 5 THE TEAM, INVESTMENT CONTRACTS AND EQUITY

By early 2004, we had presented several versions of our business plan to the university and many investors. Our team was now myself, Carl and Jean Francois (now back in Cannes, France)—other academics dropped out as the commitment required to drive this through was becoming significant. Looking inwards at ourselves, we recognised that our team was still lacking an apparent commercial edge, i.e. one “academic” engineer and two recent post‐doctoral engineers, albeit that all three of us had worked in industry. In late 2004, I contacted Dr. Martin (Terry) and invited him to join our team—fortunately for us, Terry had left Oxford Medical and now ran his own sales and marketing consultancy company. Terry had wide connections with leading obstetricians around the world—an attribute that would secure a rapid key opinion leader entry at many international hospitals and high‐level entry to the major foetal monitoring companies. We offered sweat equity to Terry, but our potential investors said that we could not give equity away without a cash injection. So, I went back to Terry and said we cannot do this, but Terry said no problem. He agreed with the investors to work for the proposed spin‐out 1 day/week for an agreed fee negotiating payment for shares over the first 12 months.

Contracts arrived from our investors for £500k [https://www.mobihealthnews.com/1690/monica-healthcare-neonatal-care-startup-gets-16m]—and we had 10 days to digest a 3‐in thick document. Good news came through that my application for a €150k Marie Curie EU grant to generate an abdominal fECG trigger for magnetic resonance imaging was successful. This required exactly the new technology that we were proposing to build at our spin‐out and provided the EU mobility for one of its top engineers Dr. Jean Francois Pieri, to work in the UK with us.

**FIGURE 4:**
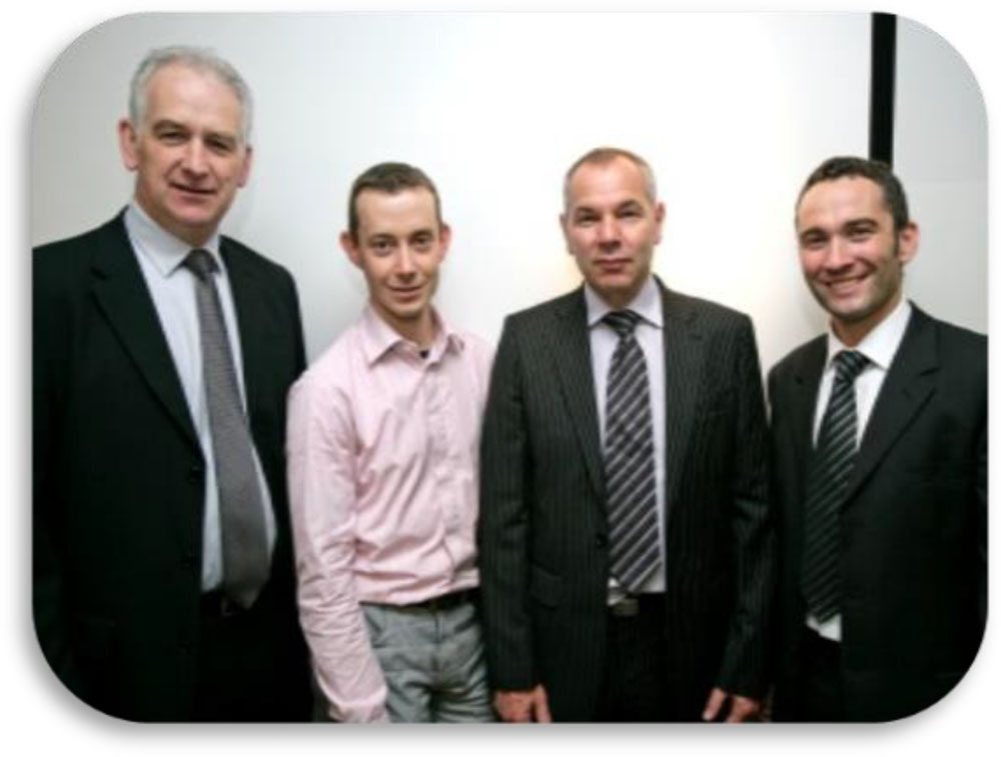
The Monica founders, Management team and Executive Directors at incorporation (2005), from left to right: Barrie R. Hayes‐Gill (CRO), Jean Francois Pieri (CTO), Terry Martin (CMO) and Carl Barratt (CEO)

So, we now had a quality team (see Figure 4), with £500k investment, a €150k EU grant, significant personal investment, four patents, a university pipeline agreement, over 500 recordings, FHR success rate increasing with each version of the device with highly supportive investors, and university. We were now ready to start an exciting but unknown journey!

## 6 COMMERCIALISATION PHASE

On the 28th April 2005, we incorporated Monica Healthcare Ltd. Our management team and executive directors consisted Carl as CEO, myself as CRO, Jean Francois as CTO and Terry as CMO. Within one month, on the 24th May 2005, we signed the investment agreement with our investors for £500,000. Our location was BioCity, the ex BASF/Boots site in the centre of Nottingham home to many other incubator companies. Our offices were next to the regional Medilink group, who were tremendously supportive with small grants, healthcare network access and publicity.

We had a two‐year, milestone‐driven, business plan to achieve CE mark for our first product AN24, an antenatal device condensing all of our university research and development into a mobile‐phone‐size device with a Bluetooth wireless link and a high‐density internal 2‐GB SD card. Data could be collected and processed initially off‐line to generate FHR—such processing was quickly moved to real time within the AN24 and the internal BT module allowed wireless transmission to a nearby display.

Our first regulatory trial in 2006 was to prove that our device (the AN24) could be used for overnight long‐term monitoring during the antenatal period, i.e. before labour and delivery (L&D). This time though, the trial was not on our home territory but in a foreign country in Europe. We were in Utrecht, Holland, superbly set up by Terry, engaging with the world's leading obstetrician in Professor Gerry Visser and his highly professional team. We now had to manage a trial remotely for the first time—flights to Utrecht were abundant, and we all visited time after time to ensure that our new device was managed correctly. The most noticeable difference was the modus operandi of the clinical engineering electrical safety department at that time. Prof. Visser had informed them in advance that we were due to arrive and they were to undertake incoming inspection of our AN24 device. This was not as simple as we expected, but it was amazingly quick and one that we must recognise as a stumbling block for evidence‐based UK MedTech research. The Utrecht clinical engineering department tested for auxiliary and leakage currents, etc., adhering to BS60601 standard and quizzed us on its performance. The notable difference from the UK was the speed of validation—within 8 h, our device was approved. But this was by no means a short‐circuit approach to approval—it was simply efficient and accurate with minimal competent authority administration. Approval was given for our device, and we were ready to start the trial under the approved local hospital ethics for a non‐CE approved device.

Over 120 mothers were monitored with our device over a range of gestations. A further 20 women were monitored for 1 h during labour and the FHR compared with the gold‐standard foetal scalp electrode. We had acceptable success rates of over 80% through all gestations overnight with BMI having no detrimental effect. As a blind test, we also manually and painstakingly located the fECGs from the raw AN24 data by eye for ∼ 10 min per mother. This acted as an independent test of the accuracy of our extraction algorithm—locating for each subject ∼1200 fECG complexes. An acceptable correlation of 0.85 was found compared to foetal scalp electrode.

As a team, we mastered the Medical Devices Directive 93/42 and its 2007 amendment—that early engagement and lessons learned from our hospital clinical engineering department back in 1993 held us in good stead. A series of international standards were studied, and standard operating procedures (SOPs) were developed to satisfy the ISO13485 medical devices quality standard. At one stage, whilst in Dusseldorf airport, on the way back from Medica, we were writing SOPs ready for our CE submission neatly handed out to each of us by Carl. In the summer of 2007, we submitted our full Technical File to our Notified Body for audit. With some minor non‐conformities that we corrected, we received our CE mark for the AN24—a portable FHR device for long‐term (24‐h) Holter monitoring of FHR. The device also provided maternal heart rate (MHR) and contained an accelerometer for movement context information. This in‐depth knowledge of the EU medical devices regulatory process proved to be an asset for the company allowing us to design future products with the European Directive in mind. We achieved all of this in 30 months with two full‐time (Carl and Jean Francois) and two part‐time (myself and Terry) staff.

We pressed on with our overnight monitoring application and signed up over 20 distributors throughout Europe with modest AN24 initial orders and minimum order quantities. Unfortunately, the commercial success of an overnight instrument was not as strong as envisaged. Fortunately, this was only one of three primary markets presented in our original business plan (April 2005): overnight monitoring, home‐care, and L&D. Hence, we still had market opportunities in the home‐care and L&D markets.

Significantly, one of the main messages that Terry was bringing back around 2009 from the market was the requirement for an improved L&D monitor—here, the annual market was > £250 M. Our European distributors, therefore, required contractions to give our product a chance in the market. At that point, we realised that we must accelerate the detection of the EHG (Electrohysterogram) with the AN24. The EHG is the electrical signal generated when a mother's uterus contracts during labour—hence contraction detection. Fortunately, we had trialled on earlier research work at the university the detection of the EHG using the same fECG electrodes, and it appeared to work effectively albeit only on a few subjects.

To address this, we undertook extended trials again in Utrecht and a new site at Witten (Germany) on the simultaneous detection of the TOCO and EHG signals during labour. In Witten, we had built another wonderful relationship with a young obstetrician with tremendous energy and drive. Results poured out from Witten and a series of publications followed not just at Witten [7] but also in Utrecht [8]. We had excellent correlation between TOCO and our EHG. What was more we could see that the TOCO was highly unreliable often requiring readjustment back to the Uterine Fundus (top of mum's bump) as the belt was either loose or slipped down the abdomen. In 2010, we were now in a position to add detection of contractions to our CE mark. Hence, we had not only an overnight monitoring device but also a true CTG monitor for L&D—a market mainly dominated by GEHC and Philips. We had captured all of the university IP into a CE‐regulatory‐approved product, but our biggest challenge was how best to package this product—we would not arrive at this answer until 2013.

We were relying on the patience of our investors who were extremely supportive partly because we met all of the timelines we were set and partly because they believed in our team. By 2010, we had received over £2.75M in investment (https://www.ukbaa.org.uk/catapult-venture-managers-support-monica-in-developing-new-generation-products/) and over £300k in grants.

Terry was still making connections around Europe and more eminent obstetricians followed and used the AN24. Various research projects were carried out with the device, and by 2012, we had seen over 50 journal papers published from numerous research hospitals around Europe. This provided a slow publicity for our device around the obstetric community backing up an early clinicians’ advice to get the work published in obstetric journals and gradually the message will get out. He was right!

The ultimate market we really wanted to address though was the USA where obesity was a real problem in L&D. So, again, Terry visited USA and met up with in particular Prof. Wayne Cohen then working as an Obstetrician and Gynaecologist as Chief of Obstetrics based at Sinai Hospital, New York. Prof. Cohen agreed to manage a trial in the USA to place Doppler ultrasound and abdominal fECG head to head against invasive foetal scalp and intrauterine pressure catheters—these invasive devices were part of normal care pathway in the USA for difficult labours. We monitored 74 women through L&D at three USA hospitals: Queens New York; Columbia New York; and Temple University Hospital, Philadelphia. The results were excellent and are summarised in Table 1 and published between 2011 and 2014 [2, 9, 10]. Here, we achieved the FHR success rate of 85% (versus 72% for Doppler) and the root‐mean‐square error (RMSE) accuracy of 5 bpm (12 bpm Doppler). For contractions, we achieved the success rate of 97% (67% TOCO) and the sensitivity of 89% (55% TOCO). The most significant result was MHR, and FHR confusion for Doppler was 10%, whilst for our device, it was 0.4%.

**TABLE 1:** USA FDA trial results (2011) comparing the Monica AN24 versus the traditional Doppler/TOCO CTG [2, 9, 10]

FHR	Monica	CTG
Reliability (%)	85	72
Accuracy (RMSE) (bpm)	5	12
**Contractions**		
Reliability (%)	97	67
Sensitivity (%)	89	55
**Maternal HR**		
Confusion rate (%)	0.4	10
**Foetal HR (BMI > 40)**		
Reliability (%)	86	66

Back in the UK, a significant product development that removed the reluctance for hospitals to purchase our product was the introduction of the IF24 in 2011. The IF24 was an interface device that received data from our Bluetooth transmitter and was directly hardwired to the installed base CTG machines. The IF24 input data is from the Bluetooth wireless link and replaces the wired Doppler and TOCO connections. The IF24 converted our FHR and EHG contractions data into foetal scalp ECG and TOCO signal inputs, respectively, thereby enabling usage of many features of the hospital's CTG machines such as alarms, printer, network facilities etc. The IF24 was a significant step in our USP offering.

**FIGURE 5:**
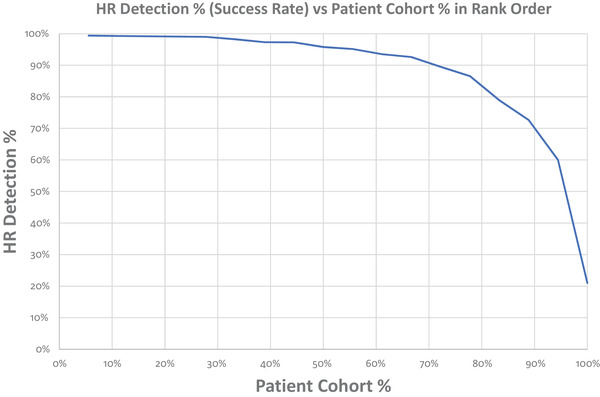
“Filling the rectangle”—graphic illustrates in the *y*‐axis the percentage success rate for each patient of a new medical device plotted against the patient in rank order (*x*‐axis). The aim of course is for 100% success in 100% of subjects; however, many medical devices achieve 90% success in 90% subjects. By improving, the product results in a gradual movement of the trace towards a rectangle

A more general key to the results presented in Table 1 was “filling the rectangle”—a term we coined when plotting the FHR success rate in rank order—see Figure 5. This plot, originally devised by Terry, shows the FHR success rate in the *y*‐axis and the *x*‐axis represents the rank order patient number, i.e. the highest patient FHR success rate is placed on the left, whilst the lowest success rate is placed at the far right. If all patients reached 100%, then we would have “filled the rectangle”. The aim was to find out why each subject did not reach at least 90%—a fact pressed on us by our highly focussed Chairman. It was never down to one feature, and often, it was a temporal issue, i.e. the foetus would move and the signal would get smaller—hence, three channels were essential. But so was lower noise although not for every subject and not for every minute. We had a list of about 10 parameters that needed addressing, and we realised that this was our onion analogy again. It is certainly a fine line between engineering common sense and an obsessive compulsive disorder, which often revealed a surprising result that one can patent.

In 2011, US Food and Drug Administration (FDA) approval followed for FHR and contractions (EHG)—FDA approvals are given K numbers and ours was “K101801”. Having once built up the confidence of the FDA in our medical device design abilities, we then included the interface IF24 (K112163) and then added in MHR & activity (K112390). By 2012, we had a fully cleared L&D CTG device to compete with the traditional Doppler CTG devices that had been in existence for the past 35 years.

**FIGURE 6:**
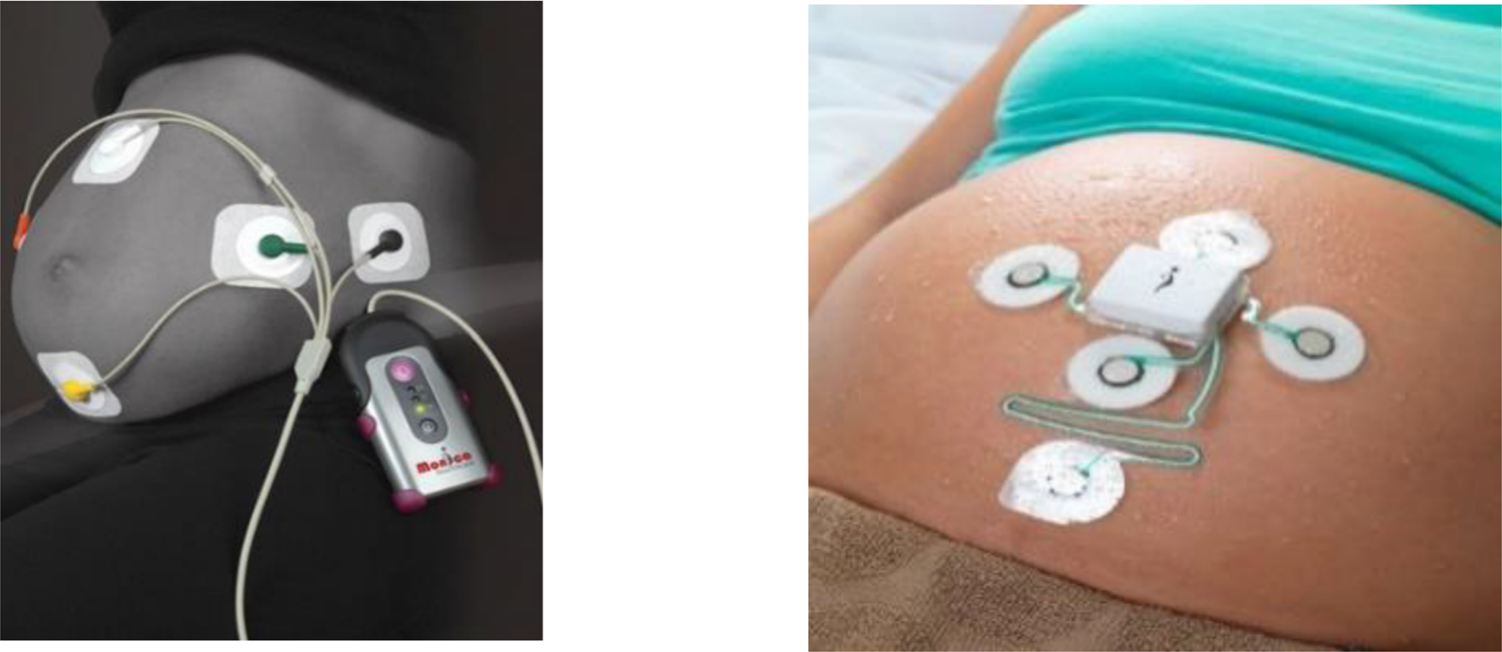
The Monica AN24 (left) with real‐time FHR extraction uses discrete electrodes. The Novii on the right shows the improved package, which incorporated all of the IP from the AN24 into the small pod (white module) but with electrodes now delivered on a single PET substrate offering a truly wearable foetal monitor

Although the AN24 had significant IP using the research generated at the university, our turnover was only £495,000 (see Companies House, 2014), but it was still missing how best to package our IP so as to offer a no choice alternative to hospitals. This came with the development of our product the Novii. This device used all of the same electronics, software and IP from the AN24 but ported this into a much smaller footprint module. This module was magnetically coupled to a PET flexible substrate (patch) that contained all of the original five electrodes of the AN24 on a monolithic PET substrate—again with driven shielding. The Novii was much simpler to administer on the mother by an easy peel and stick footprint. Figure 6 shows the comparison between the AN24 and the Novii—a single substrate, fixed electrode locations, no wires but containing all of the upside university IP of the AN24, namely, low noise, three channels, wireless, real‐time processing and mobility.

We proved the equivalence between the AN24 and the Novii substrate in an FDA USA trial. Prof. Wayne Cohen had now moved to Arizona and he undertook this trial. Wayne was a tremendous advocate of our technology, and he undertook his task with wonderful professionalism. In 2014, we received FDA 510k clearance for the Novii (K140862). Product pricing was set based upon our market appraisal, and both the AN24 and the Novii were set competitively below current CTG Doppler machines. But of course, we knew that if we increased our installed base of Novii systems, then we would sell more single‐use patches, which is where our real business model lay.

At one of our many exhibitions/trade shows, GEHC noticed our stand and eventually became our distributor in 2016. Sales and turnover grew, and we had considerable USA penetration. Philips was also our European partner via a co‐development deal (https://hitconsultant.net/2016/01/13/philips-monica-healthcare-collaborate-on-fetal-monitoring-solution/#.X70ycGj7RPY). Further trials in the USA followed (in Phoenix) that built up GEHCs confidence in our product, and by 2016, we now had a total of 12 patents (for example [6, 12]).

On the 10th March 2017, GEHC acquired Monica for an undisclosed sum with investors reaping 3.5× to 5.5× return on investment [11, 13, 14].

## 7 LESSONS LEARNED

It has been a very long but extremely enjoyable journey. With our 12 patents, a spin‐out, securing venture funding, deciphering the EU's Medical Devices Directive and navigating entry into the USA via the FDA's 510k regulatory process was a considerable achievement. Dr. Susan Huxtable, as Director of Technology Transfer, and all of the Research and Innovation team (including Dr. Bruce Venning and Dr. George Rice) at the university have been highly supportive and with us every step of the way as well as highly supportive investors.

Funding to keep the company going through many lows came from: the Stirling efforts of Carl to expertly pitch for eight investment rounds; author experience in managing IP portfolio and applying for non‐diluting grants, including GRD, SBRI, Medilink, Innovate, NTECH, Medici Fellowship awards (Carl and myself) and KTI awards; and the engineering excellence of Jean Francois in responding to Terry's astute market surveillance, trial management and distributor management that generated sales to keep the company ticking over and the investors engaged eventually leading to the development of the Novii product.

The sale of AN24s as research devices providing raw abdominal foetal electrophysiological recorders allowed research centres to publicise via over 150 journal papers (by 2021) on the usage and functionality of the Monica technology (see [15, 16, 17]).

It was interesting to note that, however, great the IP is, then it is toothless if it is not packaged correctly, and developing both the interface and a wearable patch neatly enabled this market penetration.

The success of the Monica products and the recent awards of the IET Healthcare Innovation and the RAE Colin Campbell Mitchell awards has been tremendously satisfying to the whole team. But it is just this, a true team effort, and without all parties pulling together and recognising the importance of the academic founder and the university, then we would certainly not have arrived at this success.

I would like to think that, 30 years on, we have also helped foster a culture where PhD researchers and professional engineering colleagues within our universities share a passion for the translation of technological discoveries into commercial applications for the benefit of society.

Finally, I sincerely hope that the two tragic early deaths in my life will be looking down on us with pride at what a group of university academics with a professional team can achieve in today's university technology transfer world.
